# The HIVToolbox 2 Web System Integrates Sequence, Structure, Function and Mutation Analysis

**DOI:** 10.1371/journal.pone.0098810

**Published:** 2014-06-02

**Authors:** David P. Sargeant, Sandeep Deverasetty, Christy L. Strong, Izua J. Alaniz, Alexandria Bartlett, Nicholas R. Brandon, Steven B. Brooks, Frederick A. Brown, Flaviona Bufi, Monika Chakarova, Roxanne P. David, Karlyn M. Dobritch, Horacio P. Guerra, Michael W. Hedden, Rma Kumra, Kelvy S. Levitt, Kiran R. Mathew, Ray Matti, Dorothea Q. Maza, Sabyasachy Mistry, Nemanja Novakovic, Austin Pomerantz, Josue Portillo, Timothy F. Rafalski, Viraj R. Rathnayake, Noura Rezapour, Sarah Songao, Sean L. Tuggle, Sandy Yousif, David I. Dorsky, Martin R. Schiller

**Affiliations:** 1 School of Life Sciences, University of Nevada Las Vegas, Las Vegas, Nevada, United States of America; 2 Department of Medicine, University of Connecticut Health Center, Farmington, Connecticut, United States of America; Indian Institute of Science, India

## Abstract

There is enormous interest in studying HIV pathogenesis for improving the treatment of patients with HIV infection. HIV infection has become one of the best-studied systems for understanding how a virus can hijack a cell. To help facilitate discovery, we previously built HIVToolbox, a web system for visual data mining. The original HIVToolbox integrated information for HIV protein sequence, structure, functional sites, and sequence conservation. This web system has been used for almost 40,000 searches. We report improvements to HIVToolbox including new functions and workflows, data updates, and updates for ease of use. HIVToolbox2, is an improvement over HIVToolbox with new functions. HIVToolbox2 has new functionalities focused on HIV pathogenesis including drug-binding sites, drug-resistance mutations, and immune epitopes. The integrated, interactive view enables visual mining to generate hypotheses that are not readily revealed by other approaches. Most HIV proteins form multimers, and there are posttranslational modification and protein-protein interaction sites at many of these multimerization interfaces. Analysis of protease drug binding sites reveals an anatomy of drug resistance with different types of drug-resistance mutations regionally localized on the surface of protease. Some of these drug-resistance mutations have a high prevalence in specific HIV-1 M subtypes. Finally, consolidation of Tat functional sites reveals a hotspot region where there appear to be 30 interactions or posttranslational modifications. A cursory analysis with HIVToolbox2 has helped to identify several global patterns for HIV proteins. An initial analysis with this tool identifies homomultimerization of almost all HIV proteins, functional sites that overlap with multimerization sites, a global drug resistance anatomy for HIV protease, and specific distributions of some DRMs in specific HIV M subtypes. HIVToolbox2 is an open-access web application available at [http://hivtoolbox2.bio-toolkit.com].

## Introduction

There is enormous interest in studying HIV pathogenesis for improving treatment of HIV patients. Currently, most drug therapies specifically target HIV proteins. In fact, HIV infection and replication involves ∼24 processed HIV proteins and thousands of host proteins [1]–[9]. As the study of HIV enters its fourth decade, HIV infection has become one of the best-studied systems for understanding how a virus can hijack a cell.

There is now abundant information about HIV protein sequence, structure, function, and evolution. Several databases have emerged that focus on select specific domains of HIV knowledge. From the sequence perspective, the use of sequencing and genotyping as a clinical diagnostic has driven the sequencing of tens of thousands of HIV variants, many of which are collected into databases including the Los Alamos HIV Sequence Database [10], [11]. The Protein Data Bank contains more than 1,300 HIV protein structures. And the National Institute of Standards and Technology (NIST) HIV structural database provides several tools for searching HIV drugs and their interactions with proteins [12], [13]. These tools allow investigation of drug binding sites. Since HIV has a high mutation rate, many known mutations result in drug-resistant HIV strains. These mutations have been collected into several databases updated in annual reports by the International AIDS Society [14]–[18].

Several data sources focus on a functional perspective. The HIV Human Protein Interaction Database lists many protein-protein interactions with, and posttranslational modifications of, HIV proteins. More interactions have been identified in affinity capture mass spectrometry experiments [19]–[21]. Multiple high-throughput RNAi screens have identified more than 2,400 host dependency factors (HDFs) involved in HIV replication [2]–[9]. And BioAfrica and the Los Alamos HIV Sequence Database have several additional tools for assessing different aspects of HIV function [1], [10].

Although scientists have accumulated a large amount of data regarding HIV proteins, the use of this data by researchers is limited by graphical user interfaces generally geared toward a focused facet of HIV virology. To address this issue, our laboratory recently released HIVToolbox, a database featuring integrated information about HIV proteins and a web system that presents a unified view of this information to facilitate the study of HIV sequence, structure and function [22]. In several example analyses of HIV-1 Integrase, we demonstrated that broad scale integration of sequence, structure, and functional information into a graphical mining tool can be used to identify new HIV biology [22]. Since publication of HIVToolbox, >37,000 searches have been performed.

Here, we report a number of significant updates to HIVToolbox that provide new functionality, with a general focus on antiretroviral (ARV) drugs and immune tolerance. These functions enable many new types of comparisons, which may lead to some novel global perspectives about HIV pathogenesis. Our observations include an anatomy of drug resistance in HIV protease where specific types of drug resistance mutations are localized to specific regions, and many posttranslational modification and protein-protein interactions sites overlapping with multimerization interfaces in HIV proteins. Because Tat has so many overlapping functional sites, HIVToolbox2 can assist with experimental design and interpretation of experiments related to this protein.

## Results

### Classification of HIV drug resistance

We added a number of new functions in HIVToolbox2. Several are based upon HIV drug-resistance mutations. In order to compare functional data for HIV proteins to HIV drugs, we first needed a source of drug-resistance mutations. We obtained 1,571 known HIV-1 DRMs (872 for FDA-approved drugs) from the Los Alamos HIV sequence and Stanford HIV databases, the World Health Organization website, and primary literature [10], [23].Drug-resistance mutations were then consolidated into a SQL database. The literature for each mutation was re-evaluated to classify each mutation into one of seven categories (The names and summary descriptions of the seven categories are shown in **Table 1**.)

**Table 1:** Definitions for drug resistance mutation classifications.

*Type of DRM*	*Definition*
Primary	Causes resistance without any other mutations
Primary set	Two or more mutations that cause resistance only in the presence of other primary set mutation(s)
Secondary	Enhances resistance caused by a primary mutation
Resistance precursor	A mutation that has no effect on resistance, but must occur prior to another primary or primary set of mutations
Beneficial	A mutation that prevents or reduces resistance
Beneficial set	Two or more mutations that when occurring simultaneously prevent or reduce resistance

We implemented this new scheme because, as we annotated DRMs from the literature and other databases, we observed DRMs that did not fit the standard categories of major and minor [24] (Definitions for the new scheme can be found in **Table 1**.) Briefly, DRM types designated *beneficial* or *beneficial set* (for decreasing drug resistance) are colored different shades of green. Those that cause resistance, *primary* and *primary set*, are colored red and pink, respectively. Those that amplify resistance are called *secondary set* and are colored purple. The few mutations that do not affect resistance directly, but which are precursors to other DRMs, are called *precursors* and are colored light blue. There is a checkbox option to view *ambiguous* mutations, which are colored white. Ambiguous mutations are those DRMs identified from another database for which a published peer-reviewed source could not be identified.

The combined information from the Stanford Drug Resistance database and the 2011 update from the International AIDs Society contains 188 DRMs that were classified as major or minor and had an identifiable published reference in a peer-reviewed paper (**Table 1**) [15], [16]. Review of the drug resistance literature identified a number of mutations in these databases that did not have an identifiable peer-reviewed paper; these were classified as ambiguous and not used. We also identified mutations that were published and not present in these databases. Our refactored database contained 671 unique DRMs in the seven categories discussed above (**Table 1**). Our new classification scheme is used in several new features added in the HIVToolbox2 application, and has helped to identify an anatomy of drug resistance patterns for protease and reverse transcriptase addressed later herein.

### Enhancements to the HIVToolbox2 program

HIVToolbox2 boasts many improvements over the original HIVToolbox [22]. The introduction page contains new HIV protein and drug-selection menus. The Drug menu enables direct loading of structures of HIV protein:ARV drug complexes. The HIVToolbox2 interface can also be accessed from hyperlinks from structures of HIV proteins in the Protein Data Bank website [12].

**Figure 1:**
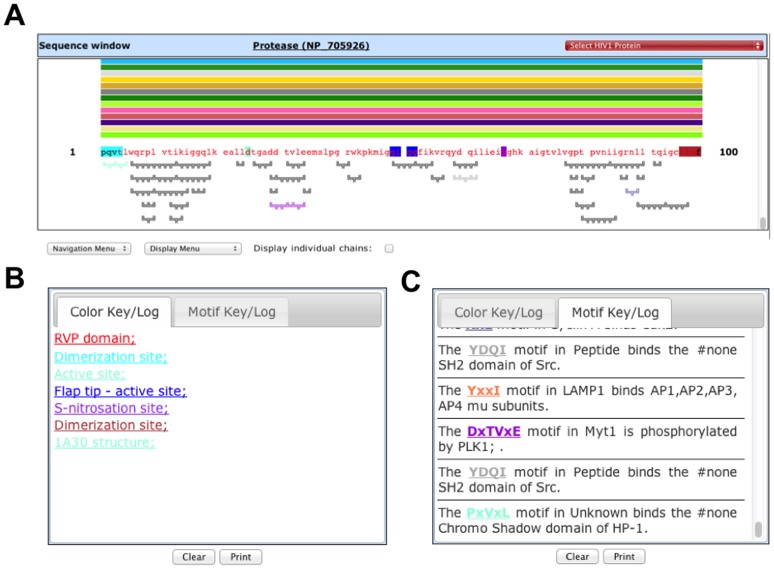
Sequence display and log windows. **A**. The Sequence window shows the sequence of the selected proteins with fonts colored by domain. Highlighted residues are for functional sites shown in the Color Key/Log window (**B**), which has hyperlinked entries. The PDB structure identifier is also shown here. Colored thick lines above the sequence show the residue mapping of different PDB structures onto the sequence. These can be selected to load different structures. A checkbox at the bottom enables display of individual chains. Figures under the sequence are for predicted or known minimotifs, which can be selected to display in a Structure window. The DxTVxE minimotif is selected and colored purple here. All hyperlinked information about each minimotif is shown in the Motif Key/Log window tab (**C**).

Once a protein or drug is selected, this directs the user to an interactive results page containing a set of windows. HIVToolbox2 has Sequence and Log windows that are similar to the original HIVToolbox with minor modifications to improve usage (**Fig. 1**). The Sequence window has been widened to show rows of 100 residues (**Fig. 1A**). The lines above the protein sequence are used to identify (hover mouse over the line) and load different structures into the structure windows. This is necessary, since many different structures and chains are available for certain HIV proteins. Two options for viewing chains are now available. The default view is visible when the “Display individual chains” checkbox is checked. This view shows all chains available for a particular structure for the selected HIV protein. Deselect this checkbox and only the structures of HIV:ARV complexes are shown, with the longest version of the chain for each structure and no chain redundancy (The lines are thicker to distinguish between the two displays). Other interactive functions of the Sequence window have not changed.

When selections are made in the Sequence window, relevant information is output to a modified Log window with two tabs. The Color Key and Motif Key log windows from the original HIVToolbox have been combined into separate tabs of a consolidated Log window (**Fig. 1B and 1C**). All minimotifs functional sites, and protein-protein interactions in the Log window are hyperlinked to PubMed abstracts for the reference sources.

A signature feature of the original HIVToolbox was three synchronized interactive protein structures displays, each showing different information about protein multimerization, domains, minimotifs, protein-protein interaction sites, functional sites, and protein sequence conservation. These windows still have the same function with some minor modifications. Protein chains are now selected from a pulldown menu in the Structure Windows title bar. This allowed us to enable the option to also select from chains and to select a drug as a wireframe model for those structures of a protein:ARV drug complex.

In HIVToolbox2, we have added three new additional synchronized interactive structure displays for viewing drug resistance mutations (DRMs), drug binding sites, and immune epitopes. As with the other three structural displays, a mouse can be used to rotate or zoom, in addition to revealing the identification of the atom by hovering the mouse cursor over any region of the protein structure. A mouse right click reveals a menu with JSmol commands and the option to open a JSmol console. All six structure displays are synchronized and interactive using JSmol commands.

**Figure 2:**
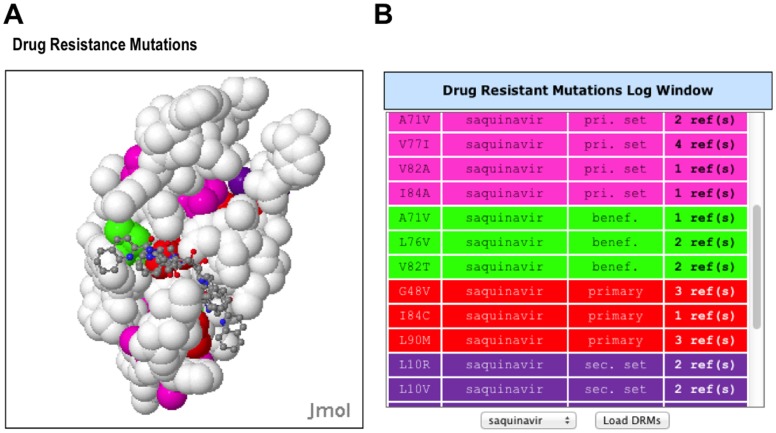
Drug Resistance Mutations structure window and table. **A**. DRM structure window showing the structure of HIV protease:Saquinavir complex (1C6Z) with DRMs for Saquinavir colored. The coloring scheme for the DRMs is beneficial (green), beneficial set (dark green; not shown), primary (red), primary set (pink), secondary set (purple) **B**. Information for each DRM is shown in a table that is color coded using the same DRM coloring scheme. DRMs for different drugs can be loaded using the pulldown menu at the bottom of the table. This sortable table also provides the chain:position, mutated amino acid, and links to the abstracts of PubMed papers supporting the DRM. The first column of this table is interactive, where a mouse click identifies the amino acid in the structure of the DRM structure window (**A**).

The new Drug Resistance Structure window (**Fig. 2A**) is initially loaded with a default structure for each protein:ARV complex, if one exists in the PDB. The DRMs in the drug resistance display are colored by a new DRM classification scheme (**Table 1**) where red  =  primary (a DRM that can cause observable resistance by itself), pink  =  primary set (a group of mutations that can cause resistance when the occur together), green  =  beneficial (a mutation that increases drug susceptibility), dark green  =  beneficial set (a set of mutations that together increase drug susceptibility), and purple  =  secondary set (which is one or more mutations that can enhance resistance when combined with a primary or primary set of mutations).

The Drug Resistance Mutation display also has a drop-down selection menu that allows selection of DRMs for a single drug to be displayed (**Fig. 2A**). The known DRMs are listed in the Drug Resistance Mutation log window with their position, drug, mutation, classification type, and hyperlink(s) to primary reference(s); rows are colored by resistance classification type. The table is interactive, where selecting the DRM identifies the location of the mutation in the Drug Resistant Mutation window with a temporary flash. Concurrently, the DRM is centered and zoomed to show the DRM (**Fig. 2A**). The DRMs for all ARV drugs are shown upon the initial loading of protein selected from the menu. A menu selector can be used to select a specific drug, and Load DRM button at the bottom of the Table enables loading of the selected ARV drugs.

**Figure 3:**
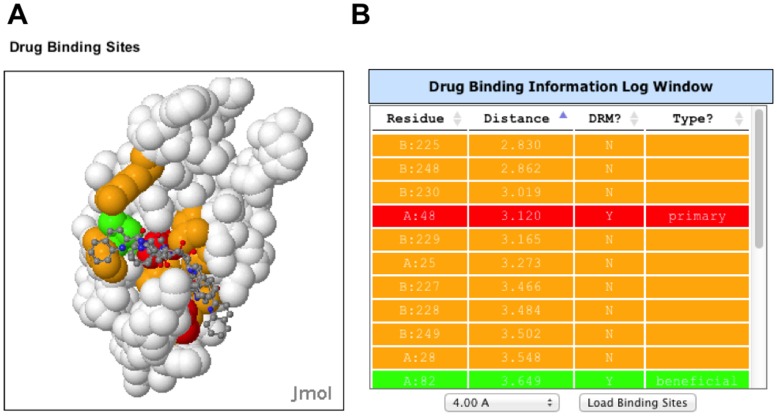
Drug Binding Site structure window and table. **A**. Drug Binding Site structure window showing the structure of HIV protease:Saquinavir complex (1C6Z) with drug binding site for Saquinavir colored. The coloring scheme for the DRMs is as in **Fig. 2** with an additional color for binding site residues that do not have a known DRM (orange). **B**. Information for each Drug Binding Site Residue is shown in a table that is color-coded using the same coloring scheme. A distance threshold between atoms of the drug and atoms of the protein (2.5–4.0 Å) can be set using a pulldown menu; 4.0 Å was set in this figure. This table provides the chain:position of the amino acid, distance, whether it is a DRM, and the type of DRM. The first column of this sortable table is interactive, where a mouse click identifies the amino acid in the structure of the Drug Binding Site window (**A**).

The new Drug Binding Sites structure window shows a surface plot with drug-binding site residues (**Fig. 3A**). The residues are colored like the DRMs, except that contact residues, for which there are no known drug resistance mutations, are colored orange. The drug is shown as a wireframe figure. A distance threshold can be selected from a pulldown menu below the Drug Binding Site Log window and then loaded (**Fig. 3B**). This threshold is for residues with an atom that makes contact with an atom of a bound drug within a specific distance. The distance threshold can be varied between 2.75 Å and 4.0 Å in 0.25 Å increments. The Drug Binding Site Log window shows the protein chain and position, distance to the closest atom in the drug, whether it is a known DRM, and the DRM classification type. Each row is colored by the class of DRM. Selection of the residue in the table shows the location of the residue in the structure window with a temporary flash, and also re-centers and zooms the structure to show the binding site residue.

**Figure 4:**
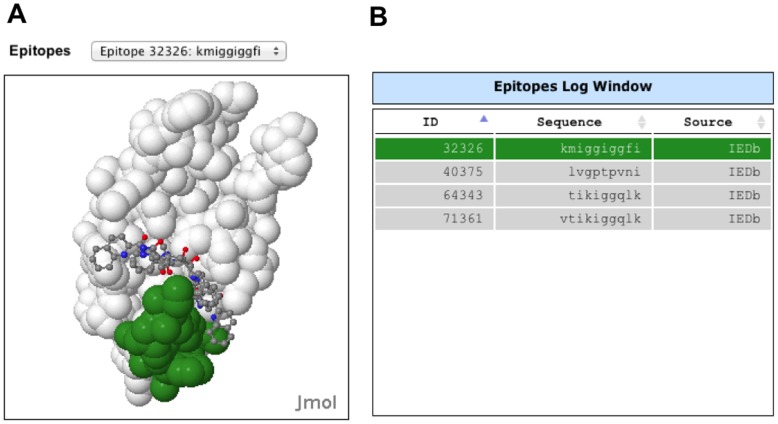
Epitope structure window and table. Epitope window showing the structure of protease:Saquinavir complex (1C6Z) with immune epitope KMIGGIGGFI colored green. Different positive immune epitopes for the loaded HIV protein from the IEDB can be selected using a pulldown menu on the top of the window that shows the IEDB id number and peptide sequence or from the sortable Epitopes Log table [25].

The new Immune Epitope structure window has positive immune epitopes colored on the surface of an HIV protein structure (**Fig. 4A**). Immune epitopes and their identifiers from the HIV Immune Epitope database 2.0 can be selected from a pulldown menu above the window or by selecting the epitope from the Epitopes Log window (**Fig. 4B**) [25]. If the shift key is held down while selecting multiple epitopes from the log window, multiple epitopes can be shown concurrently. The table also has the epitope ID and hyperlink to the entry in the Immune Epitope Database.

**Figure 5:**
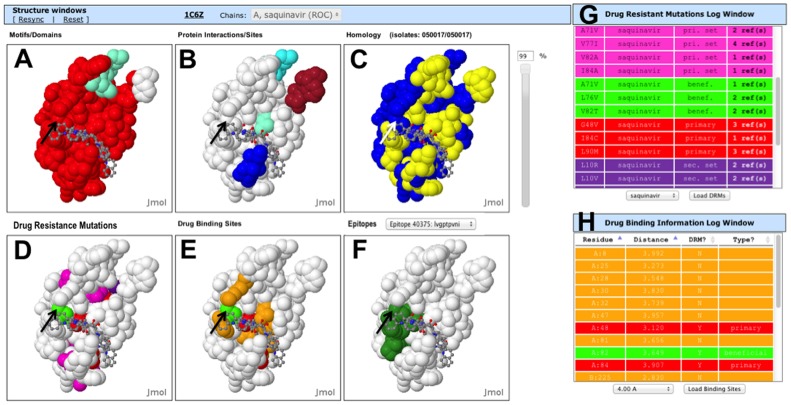
HIVToolbox2 structure windows for the HIV protease:Saquinavir complex. Synchronized structure windows of HIV protease:Saquinavir complex (1C6Z; chain A; **A**–**F**) and information tables (**G**–**H**). The coloring schemes are: **A.** Domains and motifs are colored in the Domain/Motif window as defined in the Log windows (not shown). **B.** Functional sites and protein-protein interactions are colored in the Protein Interactions/Sites window **C.** Conservation of the residues is shown in the Homology window. The conservation slide threshold is set to 99% amino acid identity and yellow residues are conserved among 50,017 viral sequences shown here. **D.** DRM window with DRMs for Saquinavir colored. The coloring scheme for the DRMs is beneficial (green), beneficial set (light green), primary (red), primary set (pink), secondary set (purple) **G.** Information for each DRM is shown in a table that is color coded using the same DRM coloring scheme. DRMs for different drugs can be loaded using the pulldown menu at the bottom of the table. This table also provides the original amino acid, position, mutated amino acid, and links to the abstracts of PubMed papers supporting the DRM. The first column of this table is interactive, where a mouse click identifies the amino acid in the structure of the DRM window (**D**)**.** **E.** Drug Binding Site window showing the structure of protease with the binding site for Saquinavir colored. The coloring scheme for the DRMs is as in **Fig. 2** with an additional orange color for binding site residues that do not have a known DRM (orange). **H.** Information for each Drug Binding Site Residue is shown in a table that is color-coded using the same coloring scheme as in **E**. A distance threshold between atoms of the drug and atoms of the protein (2.5–4.0 Å) can be set using a pulldown menu; 4.0 Å was set in this figure. This table provides the amino acid position, shortest distance to a drug atom, whether it is a DRM, and the type of DRM. The first column of this table is interactive, where a mouse click identifies the amino acid in the structure of the Drug Binding Site window (**E**)**. F.** Epitope window showing protease with the immune epitope KMIGGIGGFI colored green. Different positive immune epitopes for the loaded HIV protein from the IEDB can be selected using a pulldown menu on the top of the window that shows the IEDB id number and peptide sequence^4^.

The six interactive structural displays are organized for direct comparison (**Fig. 5A**–**F**). These are interactive with the three adjacent log windows (**Fig. 5G,H**; the Epitopes Log window that is not shown here). This layout facilitates interpretation of data in the context of structure, function and sequence conservation. The new structure windows in HIVToolbox2 provide a new means to study HIV pathogenesis, and relations to immonology.

Several data items in the HIVToolbox2 database have been updated (**Table 2**). We have added additional sequences from the 2012 Los Alamos HIV Sequence database [10]. The HIVToolbox2 database now contains ∼502,000 HIV protein sequences from different patient blood samples. HIVToolbox was updated and now contains ∼1200 structures of HIV proteins, including several new structures of protein:ARV drug complexes from the PDB [12]. We calculated all residues in HIV protein that were within 3.5 Å of an atom in the complexed molecule to create binding sites that were entered in the HIVToolbox2 database as new protein-protein interactions or for non-protein molecules as new sequence features. Some additional functions associated with sequence elements, which were identified in the literature, were added to the database. For all annotations, we now provide a hyperlink to a PubMed abstract that identified the interaction. The HIVtoolbox database is updated at least annually, which we plan to continue.

**Table 2:** HIVToolbox2 Data Statistics.

Data Type	Number
Major DRMs	155
Minor DRMs	33
Primary DRMs	186
Primary set DRMs	368
Beneficial DRMs	12
Beneficial set DRMs	21
Secondary set DRMs	83
Resistance Precursor DRMs	1
Ambiguous DRMs	274
Total non-ambiguous DRMs	671
Sequence features	316
Protein-protein interactions	1,453
Predicted and known motifs	6,373
HIV proteins (processed)	24
Structures	∼1,200
Epitopes	828
FDA approved drugs	27

### New workflows enabled in HIVToolbox2

#### Workflows #1-16

Six integrated structural viewers make it easy to compare different types of data with regard to sequence, structure, function, sequence conservation, drug resistance and immune epitopes. The 16 different types of pairwise comparisons enabled are shown in **Table 3**. Workflows 4–16 are now enabled in HIVToolbox2. One example from these 16 workflows is shown for a HIV protease:Saquinavir complex in **Fig. 5**. This example of multiple comparisons shows that the T82 residue (**arrows**) is in a region that is not conserved (panel C – blue residues are not conserved) that is outside the active site (panel B) is a beneficial mutation (panels D, G – green) that makes contact with the drug (panels E, H) and is an immune epitope #40375 (panel F).

**Table 3:** Example of use cases 1–16 enabled by HIVToolbox2.

Use case	Window Relationships	Example
1	Motif/Domains vs. Functional sites/Protein-Protein Interactions	The DNA primer binding site is in the RVT connect domain of RT.
2	Motif/Domains vs. Conservation	The RT domain has the highest conservation when compared to the thumb and connect domains.
3	Functional sites/Protein-Protein Interactions vs. conservation	Many functional and protein interaction sites in Tat are conserved in >90% of 2482 sequences
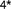	Motif/Domains vs. DRMs	The only DRM in the thumb domain of RT is the L283I beneficial set mutation for Efavirenz
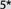	Motif/Domains vs. Drug binding sites	The Nevirapine binding site is in the RVT domain of RT
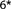	Motif/Domains vs. Immune epitopes	The entire p24 domain of capsid has immune epitopes except for residues 93–98, 100 and 220. Some are involved in inter-monomer contacts.
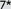	functional sites/Protein-protein Interactions vs. conservation	The S16 phosphorylation sites and K28 acetylation site are completely conserved in 2482 Tat sequences.
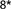	Functional sites/protein-protein interactions vs. DRMs	The S230R secondary set DRM in Integrase is a residue involved in DNA binding.
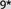	Functional sites/Protein-Protein Interactions vs. drug binding sites	Epitopes 1180, 2835,1292, 13675 and 14143 are in the RNase domain of p66 RT
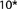	Functional sites/Protein-Protein Interactions vs. immune epitopes	Epitopes 69437, 69439, 59975 are in dATP binding site of RT.
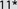	DRMs vs. conservation	When compared to ∼50,000 virus sequences, beneficial mutations N88S 2% and I50L <1%. Primary I47A <1%, I50V <1%, I54L/M<1%, I84V 3% Primary set I54V is in 88%.
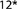	Drug binding sites vs. conservation	Most APV binding site residues are highly conserved which the exception of I84 and G48 ∼2% that later is not a primary mutation
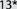	Immune epitopes vs. conservation	Epitope 32326 is highly conserved but some subtypes show modest conservation of I46 and M54
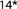	DRMs vs. Drug binding sites	Most DRMs are in residues within 4 Å of atoms in the Amprenavir drug; however there are notable exceptions of beneficial mutation N88S and several secondary mutations. There are also a number of drug binding site residues where DRMs have not been observed.
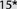	DRMs vs. Immune epitopes	For Amprenavir and protease several immune epitopes overs lab with import DRMS I84V, primary; L76V and V32I primary set are contained in epitope 40375; M46I primary set, Beneficial mutation I50L; primary, I50V or I54L/M are contained in epitope 32326
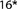	Drug binding sites vs. immune epitopes	For Amprenavir and protease immune epitopes 40375 and 32326 contain many binding site residues and also involve residues that contact the drug

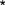
Previously not capable in the original HIVToolbox application.

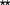
Conservation can be examined for all viruses or within subtypes.

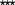
NP_705926 is used as the reference sequence for protease.

Different aspects of workflows #17-21 described below are enabled in HIVToolbox2 and were not possible with HIVToolbox.

#### Workflow #17: Predicted effectors of HIV protein multimerization

Most HIV proteins form multimers required for their activity (**Table 4**). We considered that multimerization could potentially be regulated by other functional sites in proteins. Therefore, we looked for functional sites within the multimerization interface in different structures of HIV proteins. We noticed a common pattern where phosphorylation sites were present at sites of subunit interactions in structures of Vif, Rev, Tat, and Matrix multimers [26]–[29]. We identified some protein-protein interaction sites in Nef, Rev, Vif, and Vpr that overlap with the multimerization interface. Thus, they may be involved in HIV protein oligomerization and activity [26], [27], [30], [31]. The Protein Sequence window can be used to investigate known and predicted minimotifs that overlap with HIV protein oligomerization sites.

**Table 4:** Multimerization of HIV proteins.

HIV protein	Functional multimer	Potential or Known Multimerization inhibitors	Reference
Gag	oligomer	None	[51]
Protease	homodimer	None	[52]
Reverse transcriptase	p51/p66 heterodimer	None	[53]
Integrase	homotetramer	DNA binding	[54]
Capsid	homohexamer, homotrimer	None	[29], [55], [56]
Nef	homodimer	Fyn, AP1 mu and Peroxisomal Acyl-CoA thioesterase 1	[30]
Rev	homodimer	PKCα phosphorylation site, nucleophosmin	[28]
Matrix	homohexamer	Phosphorylation sites	[29]
Nucleocapsid	monomer	None	[57]
Tat	dimer	Phosphorylation and acetylation sites	[26], [58]
GP41	GP41/GP120 heterohexamer	Enfuvirtide	[59]–[61]
GP120	GP41/GP120 heterohexamer	Enfuvirtide	[59]
Vpr	homodimer	TATA Box binding protein, p6	[31], [62], [63]
Vpu	monomer	None	[64]
P6	monomer	None	[62], [65]
Vif	homodimer, homotrimer	Phosphorylation sites, Vasopressin activated calcium mobilizing receptor 1 binding site	[27], [66]

*Bolded residues are known multimerization inhibitors.

#### Workflow #18: Identification of overlapping or non-overlapping functionalities to generate new hypotheses

**Figure 6:**
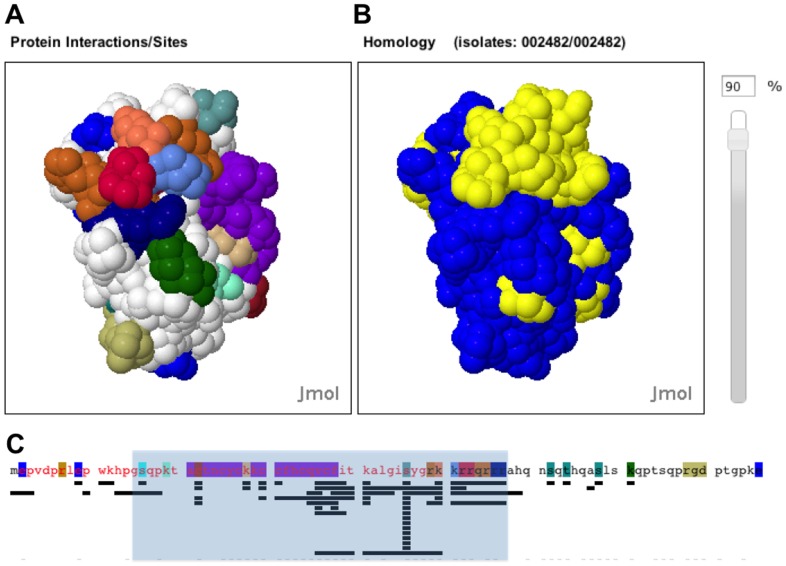
Functional sites and their conservation in Tat. Output of HIVToolbox2 for Tat. **A.** Surface plot of Tat (1TAC) with functional site amino acids colored. Colors are ADP ribosylation sites (blue), proteolysis site (cyan), dimerization site (purple), phosphorylation sites (dark brown, teal), acetylation sites (tan, orange), RNA binding site (brown), methylation sites (red, royal blue), ubiquitination site (gray), and cell attachment site (green). Other sites on the opposite face are not shown. **B.** Surface Plot showing residues >90% conserved in 2482 Tat sequences (yellow) **C.** Protein Sequence of Tat. Highlighted colors are as described in **A**. Mapping of functional site (highlighted fonts) and protein-protein interaction sites (lines underneath sequence). These lines map Tat interaction with Cyclin T1, CDK9, CDK2, Lysine acetyl transferase 2B, 5, Tat interaction protein, Transcription elongation factor 1, p53, p73, Zinc finger and BTB domain containing 7A, Early growth response 1, BCL2-like 11, Protein phosphatase 1, Tubulin α4a, TBP-associated factor 1, several PKCs, and PKD3, Histone cluster 1, Karyopherin β1, SWI/SNF-related matrix-associated actin-dependent regulator of chromatin a2, DNA directed RNA polymerase II, Eukaryotic translation initiation factor 2α kinase 2 (left to right). The blue shaded box shows residues 15–57.

Consolidation and integration of the functional information in HIVToolbox2 can facilitate experimental design and interpretation. One of the best examples of how coordination of data can be used to generate new hypotheses comes from examination of Tat with HIVToolbox2 (**Fig. 6**). The HIV Tat transcription factor is a potential drug target [32]. Examination of the Tat sequence shows a functional hotspot between residues 15–57 (**Fig. 6C** **,** blue shaded box). In this region, there are binding sites for ∼30 different proteins and multiple types and sites of posttranslational modifications (PTMs). These residues are some of the mostly highly conserved regions in Tat (**Fig. 6B**). There are several examples in this region of Tat where functional sites are known to compete with each other [33].

Structure mapping of sites on Tat with HIVToolbox2 (**Fig. 6A**) allows evaluation of which proteins or PTMs have residues that overlap other sites. These are expected to be competitive functions, in many cases. Several previously unknown examples of such functional overlaps are easily recognized. The Cyclin T1 and CDK9 binding sites overlap with an ADP ribosylation site. Tat also binds p53, which overlaps with several sites (Karopherin beta, Proteosome alpha 1, and DNA directed RNA polymerase II binding sites, as well as RNA binding site, and protein methylation sites and acetylation sites). From a compatibility perspective, the p53 and TBP associated factor 1 binding sites are adjacent to, but don't overlap with, the Tat dimerization site and Cyclin T binding sites. However, the TBP and p53 do have overlapping residues. There are far too many combinations to discuss here. But clearly, this tool is a source for better understanding the multiple roles of Tat. HIV2Toolbox2 helps interpret results as demonstrated by examining the hot spot region of Tat.

#### Workflow #19: Known and predicted minimotifs in HIV proteins

HIV Rev binds the Rev Response Element (RRE) in the HIV RNA genome and facilitates transport of the genomic RNA from the nucleus to the cytosol. Rev has known sequence elements associated with dimerization, phosphorylation, methylation, RNA binding, and ubiquitination. We examined Rev for minimotifs to demonstrate the utility of this type of workflow. The region of Rev between P76-L83 seems to be multifunctional, binding four different proteins. This region is not in the dimerization site or other functional sites. This region of Rev binds ArfGAP, a protein involved in nuclear export [34]. The nuclear export function seems to have redundancy with an overlapping NLP1 binding site, which serves as a bridge protein to bind Exportin 1 for nuclear export [35]. These are consistent with the known roles of Rev in export of the genomic HIV. This region also binds to prothymosin α, a protein involved in transcription, and Sam68, another RNA binding protein that is involved in HIV genomic RNA export, as well as in translational regulation of HIV RNA [36]. Given that there are four different binding proteins for this site, and that Rev forms dimers, it is currently unclear if Rev forms heterotetramers with two of its binding partners, and, if so, with which pairs of proteins. This is may be an important facet of Rev function.

#### Workflow #20: Global resistance landscapes

**Figure 7:**
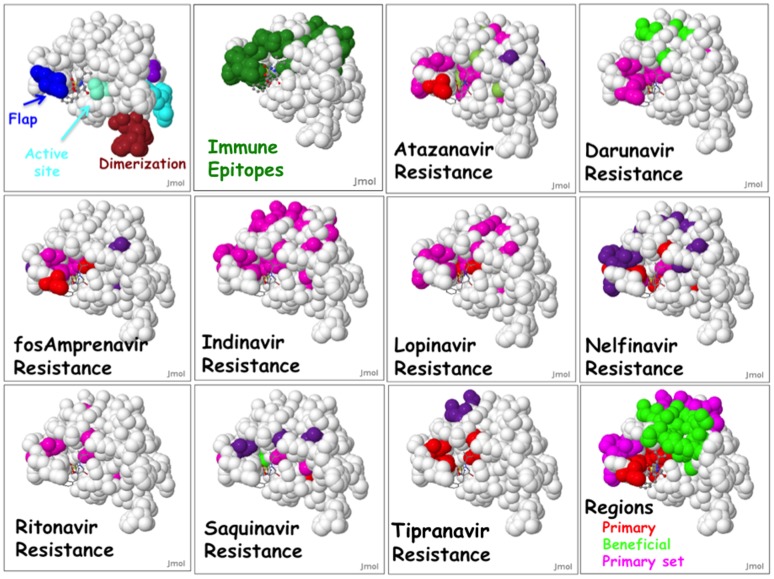
Protease DRM landscape. A collection of DRM surface plots for HIV protease generated with HIVToolbox2. All plots are for a structure of Amprenavir (ball and stick) bound to one subunit of protease (1HPV, chain A). The top-left panel shows functional sites and the adjacent panel shows all known immune epitopes from the IEDB ids 32326, 40375, 64343, and 71361. All other panels show resistance to different FDA-approved HIV protease inhibitors. The last panel shows a compendium of DRMs identify regions of the protease with different types of DRMs. The coloring of DRMs is as in **Fig. 2**.

As an example of a global resistance landscapes, we examined HIV protease inhibitors using HIVToolbox2 (**Fig. 7**). This type of analysis demonstrates the utility of both the new DRM classification scheme and the HIVToolbox2 tool. When we examine the distribution of the DRMs on the protease surface plots for all FDA approved drugs that target HIV protease, several resistance patterns become apparent. All known primary mutations are in the drug-binding pockets of the drugs. Primary set mutations contain residues that are either in the binding pocket or immediately juxtaposed, but only on one face of the protease. Beneficial or beneficial set mutations are clustered near the active site but in a region overlapping with the primary set mutations. Secondary-set mutations generally overlap with a region containing primary set mutations. Mutations are observed in the active site and in residues that form a flap covering the active site, but never in the dimerization residues. The active site, flap, and dimerization site residues are highly conserved, whereas many residues in the primary set and beneficial regions have lower conservation levels (as little as 85% in ∼50,000 HIV-1 protease sequences).

#### Workflow #21: Examining amino acid frequencies by HIV subtype

A useful feature of HIVToolbox2 is that it enables the ability to view mutations and their frequencies in specific viral subtypes. This can be accomplished for any known amino acid in an HIV protein by using the pulldown menus at the bottom of the Sequence window, selecting the Clustal Alignment in the Sequence Alignment section, and then selecting the PSSM. The frequencies are calculated from the data in the Los Alamos HIV Sequence database, which features data that is not collected in a single standardized epidemiological study, but does provide a rough snapshot of mutation prevalence in each subtype.

To show the utility of this tool, we examined the beneficial and primary DRMs for HIV drug resistance in protease (**Table 5**). In this analysis, we used NP_705926 as the reference sequence. Some interesting patterns were apparent. The L10V Beneficial set DRM for Atazanavir is prevalent in the F1 subtype, but this must occur with L24I, which is only in 4% of the Subtype F1 sequences. The K20I beneficial DRM for Darunavir is in most of the 612 subtype G sequences. Although this was previously known as a beneficial mutation, it was not known to be prevalent in Subtype G viruses [37]. The V82A beneficial DRM for Darunavir and beneficial set for Atazanavir [37]–[39] is prevalent in the B and F1 subtypes (19–25% of sequences). The M46L is also abundant in subtype B. This type of subtype analysis can also be performed for any minimotif, functional site, immune epitope, protein-protein interaction, and drug binding site residue with HIVToolbox2.

**Table 5:** HIV Mutation distributions for protease in select HIV-1 Class M subtypes.

DRM*	A	A1	B	C	D	F1	G	Total
N	562	1507	34086	3052	781	337	612	49998
**Beneficial or Beneficial set DRMs**								
L10V	5	5	6	1	6	29	1	5
K20I	10	4	0	1	1	1	94	4
L24I	0	0	2	0	1	4	0	2
E35D	87	90	9	20	23	93	49	21
M36I	98	98	14	80	52	93	98	30
I50L	0	0	0	0	0	0	0	0
I54L	0	0	0	0	0	0	0	0
A71V	1	0	12	1	1	5	4	10
L76V	0	1	1	2	1	2	1	1
V82T	0	0	0	0	0	1	3	1
V82A	1	0	25	1	1	19	1	16
V82F	0	0	1	0	0	1	0	2
N88S	0	0	0	0	0	1	0	2
**Primary DRMs**								
D30N	0	0	2	1	1	3	1	1
L33F	1	1	0	0	1	3	0	0
M46I	1	1	7	2	1	8	4	5
M46L	1	0	15	0	1	6	2	8
I47A	0	0	0	0	0	0	0	0
I47V	0	0	0	0	0	0	1	0
G48V	0	0	7	0	0	3	0	4
I50L	0	0	0	0	0	0	0	0
I50V	0	0	0	0	0	1	0	0
I54A	0	0	0	0	0	0	0	0
I54L	0	0	0	0	0	0	0	0
I54M	0	0	0	0	0	1	0	0
Q58E	1	0	0	0	1	4	1	1
T74P	0	0	0	0	0	1	0	0
L76V	0	1	1	2	1	2	1	1
V82A	1	0	25	1	1	19	1	16
V82F	0	0	1	0	0	1	0	2
V82L	0	0	0	0	0	0	0	0
V82S	0	0	0	0	0	1	0	0
V82T	0	0	0	0	0	1	3	1
I84A	0	0	0	0	0	0	0	0
I84C	0	0	0	0	0	0	0	0
I84V	1	0	1	1	1	1	1	3
N88S	0	0	0	0	0	1	0	2
M89I	2	1	0	3	1	6	6	1
M89V	0	0	0	0	0	1	3	2
L90M	1	1	9	3	2	7	8	10

*The reference sequence for HIV-1 protease is NP_705926.

#### Availability, video tutorials and user guide

HIVToolbox2 is an open-access web application available at http://hivtoolbox2.bio-toolkit.com. The application has been tested on all major web browsers and operating systems. A Help page for HIVToolbox2, with a summary, funding, video tutorials, user guide, research papers and contact is at http://www.bio-toolkit.com/HIVToolbox/project. The SQL database of drug resistant mutations is available upon request.

## Discussion

Our second release of the HIVToolbox provides both data updates and new functions enabling 21 different types of workflows; only three were possible with the original HIVToolbox. As well as our previous focus on sequence, structure, function and conservation, we have added information related to HIV pathogenesis: HIV drugs, drug resistance and immune epitopes. By using HIVToolbox2 to explore some of these workflows, we have identified some interesting aspects of HIV proteins that become more obvious once all the data is integrated and visualized. These include the following findings: (1) almost all HIV proteins form homomultimers; (2) host proteins bind or covalently modify interfaces of HIV protein homomultimeration; (3) HIVToobox2 helps with interpretation of complex interaction interfaces in proteins like Nef and Tat; (4) a protease drug resistance landscape reveals a distinct resistance anatomy; and (5) some DRMs are much more prevalent in some subtypes.

### HIV protein multimers

Although multimerization has been studied for individual HIV proteins, our consolidation of data for HIV structures has helped emphasize that most HIV proteins form some type of homoligomers. To our knowledge, this has not been previously reviewed. Protease, RT, Nef, Rev, Tat and Vif can form dimers. Env, GP120, GP41, Capsid, and Vif can from trimers, and Capsid and matrix can form hexamers (**Table 4**). Nucleocapsid, p6, and Vpu are not known to multimerize. The HIV homomultimers are, in most cases, essential for activity of the protein, and multimerization has been extensively investigated as a mechanism of inhibition of replication [40]–[47].

The other interesting aspect of HIV protein multimerization is that several posttranslational interactions and interactions with host proteins are within HIV homomultimerization interfaces and expected to compete (**Table 4**). This observation suggests that host factors may play an important role in controlling where and when HIV proteins multimerize, thus controlling their activity. This is interesting because one general approach in inhibiting HIV replication has been to generate peptides or compounds that block multimerization of key HIV proteins [40]–[47].

### Tat interpretation

As knowledge of protein function grows, it becomes clearer that some regions of proteins are very complex. For example, a hotspot of interaction has been identified in HIV Nef [48]. In integrating data, this becomes apparent for Tat, where there are over 30 protein-protein interaction and posttranslational modifications in a 32 amino acid region. Many scientists model highly complex proteins in networks, where Tat and other proteins with many interactions are considered hubs. HIVToolbox2 advances the analysis of Tat as a hub protein by enabling rapid interpretation in the context of structure. The structure can be used to derive sets of rules for the hub network node that can be tested. An example of a rule that can be extracted from the HIVToolbox2 interface is “Methylation at K51 overlaps with RNA binding site, thus one rule would be that K51 methylation and RNA binding on the same Tat monomer are mutually exclusive.”

### HIV protease resistance landscape

A new feature in HIVToolbox is the ability to view DRMs mapped onto the surface of protein structures. **Fig. 7** shows a comparison of DRMs for various FDA-approved HIV protease inhibitors. This analysis, when combined with an extended DRM classification scheme, reveals an anatomy of resistance in protease. Each type of DRM is localized to a specific region of protease. Furthermore, drug resistance mutations have not yet been observed near the dimerization or nitrosylation sites. The observation of such a global pattern is not easily recognized without the visual mining enabled by HIVToolbox2. We note that the region covered by 4 protease immune epitopes is inclusive of the regions that have primary and primary set mutations. This resistance anatomy may prove useful for pharmaceutical companies in designing future ARVs that are less susceptible to drug resistance.

### DRM prevalence in HIV-1 subtypes

The original HIVToolbox had a function to look at sequence from blood samples for different HIV subtypes. By including DRMs in HIVToolbox2, we could now examine how different DRMs were distributed among different HIV subtypes. These observations must be considered with caution, as the sequence data were not collected as a single epidemiological study, but rather are a compendium of many different studies and samples. Nevertheless, there were some interesting observations (Workflow 21, **Table 5**). The V82A DRM, which is beneficial for Darunavir and part of a beneficial set for Atazanavir, was in 19–25% of subtype B and F1 samples [37], [38], [49].

## Conclusions

HIVToolbox2 updates the original HIVToolbox with new data, new functions and improved ease of use. Data integration and the new functions enable many new types of workflows that have resulted in several new global observations: (1) most HIV proteins form higher order homomultimers; (2) many multimerization interfaces have posttranslational modifications or protein-protein interactions that may compete with or enhance multimerization; (3) HIV protease has a global resistance anatomy; (4) protein structure can be used to help examine network hub proteins such as Tat; and (5) some DRMs are more prevalent in specific Class M subtypes.

## Methods

### Software engineering

HIVToolbox2 was built as a standard, three-tier J2EE web application consisting of 1) an underlying relational MySQL database, 2) a set of standard Java data access objects that pull data from the database, and 3) a set of dynamic interactive web pages. Several classes were translated from Java to JavaScript so that the structure interaction interface is generated on the client side, instead of the server side. This is better suited to cross-browser and cross-platform compatibility.

### Data sources

HIV-1 data from external sources such as the Protein Data Bank, NCBI, Los Alamos HIV sequence database, etc. was collected, curated, and stored in the HIVToolbox2 database. The HIVToolbox2 database has ∼502,000 total sequences for HIV blood samples from 126 different countries [22]. These sequences were derived from nucleotide sequences from the Los Alamos HIV sequence database, which were converted into amino acid sequences using BioJava 3.03 (http://www.biojava.org).

### Distance and frequency calculations

In order to identify amino acids that contact atoms in the drug we used BioJava. Distance thresholds were set from 2.5–4.0 Å in 0.25 Å increments. The pre-calculated distance data is stored in MySQL tables and returned upon client requests. The residue frequencies were calculated from multiple sequence alignments as previously done using ClustalΩ for clade specific alignments in the HIVToolbox database [50]. The pre-processed data for the frequency of amino acids for DRMs are stored in a MySQL table.

## References

[1] Doherty RS, De Oliveira T, Seebregts C, Danaviah S, Gordon M, et al (2005) BioAfrica's HIV-1 Proteomics Resource: Combining protein data with bioinformatics tools. Retrovirology 2: 18. 15757512 10.1186/1742-4690-2-18 PMC555852

[2] Bushman FD, Malani N, Fernandes J, D'Orso I, Cagney G, et al (2009) Host cell factors in HIV replication: meta-analysis of genome-wide studies. PLoS Pathog 5: e1000437. 19478882 10.1371/journal.ppat.1000437 PMC2682202

[3] Konig R, Zhou Y, Elleder D, Diamond TL, Bonamy GM, et al (2008) Global analysis of host-pathogen interactions that regulate early-stage HIV-1 replication. Cell 135: 49–60. 18854154 10.1016/j.cell.2008.07.032 PMC2628946

[4] Zhou H, Xu M, Huang Q, Gates AT, Zhang XD, et al (2008) Genome-scale RNAi screen for host factors required for HIV replication. Cell Host Microbe 4: 495–504. 18976975 10.1016/j.chom.2008.10.004

[5] Brass AL, Dykxhoorn DM, Benita Y, Yan N, Engelman A, et al (2008) Identification of host proteins required for HIV infection through a functional genomic screen. Science 319: 921–926. 18187620 10.1126/science.1152725

[6] Yeung ML, Houzet L, Yedavalli VSRK, Jeang K-T (2009) A genome-wide short hairpin RNA screening of jurkat T-cells for human proteins contributing to productive HIV-1 replication. J Biol Chem 284: 19463–19473. 19460752 10.1074/jbc.M109.010033 PMC2740572

[7] Börner K, Hermle J, Sommer C, Brown NP, Knapp B, et al (2010) From experimental setup to bioinformatics: an RNAi screening platform to identify host factors involved in HIV-1 replication. Biotechnol J 5: 39–49. 20013946 10.1002/biot.200900226

[8] Nguyen DG, Wolff KC, Yin H, Caldwell JS, Kuhen KL (2006) “UnPAKing” human immunodeficiency virus (HIV) replication: using small interfering RNA screening to identify novel cofactors and elucidate the role of group I PAKs in HIV infection. J Virol 80: 130–137. 16352537 10.1128/JVI.80.1.130-137.2006 PMC1317519

[9] Rato S, Maia S, Brito PM, Resende L, Pereira CF, et al (2010) Novel HIV-1 knockdown targets identified by an enriched kinases/phosphatases shRNA library using a long-term iterative screen in Jurkat T-cells. PLoS ONE 5: e9276. 20174665 10.1371/journal.pone.0009276 PMC2822867

[10] Leitner T, Foley B, Hahn B, Marx P, McCutchan F, et al. (2005) HIV Sequence Compendium, 2005 Los Alamos, NM: Theoretical Biology and Biophysics Group, Los Alamos National Laboratory.

[11] Kuiken C, Korber B, Shafer RW (2003) HIV sequence databases. AIDS Rev 5: 52–61. 12875108 PMC2613779

[12] Berman HM, Westbrook J, Feng Z, Gilliland G, Bhat TN, et al (2000) The Protein Data Bank. Nucleic Acids Res 28: 235–242. 10592235 10.1093/nar/28.1.235 PMC102472

[13] Prasanna MD, Vondrasek J, Wlodawer A, Rodriguez H, Bhat TN (2006) Chemical compound navigator: a web-based chem-BLAST, chemical taxonomy-based search engine for browsing compounds. Proteins 63: 907–917. 16508960 10.1002/prot.20914

[14] Johnson VA, Brun-Vézinet F, Clotet B, Günthard HF, Kuritzkes DR, et al (2010) Update of the drug resistance mutations in HIV-1: December 2010. Top HIV Med 18: 156–163. 21245516

[15] Rhee SY, Gonzales MJ, Kantor R, Betts BJ, Ravela J, et al (2003) Human immunodeficiency virus reverse transcriptase and protease sequence database. Nucleic Acids Res 31: 298–303. 12520007 10.1093/nar/gkg100 PMC165547

[16] Johnson VA, Calvez V, Gunthard HF, Paredes R, Pillay D, et al (2011) 2011 update of the drug resistance mutations in HIV-1. Top Antivir Med 19: 156–164. 22156218 PMC6148877

[17] Johnson VA, Brun-Vezinet F, Clotet B, Gunthard HF, Kuritzkes DR, et al (2009) Update of the drug resistance mutations in HIV-1: December 2009. Top HIV Med 17: 138–145. 20068260

[18] Johnson VA, Brun-Vezinet F, Clotet B, Kuritzkes DR, Pillay D, et al (2006) Update of the drug resistance mutations in HIV-1: Fall 2006. Top HIV Med 14: 125–130. 16946457

[19] Gautier VW, Gu L, O'Donoghue N, Pennington S, Sheehy N, et al (2009) In vitro nuclear interactome of the HIV-1 Tat protein. Retrovirology 6: 47. 19454010 10.1186/1742-4690-6-47 PMC2702331

[20] Jäger S, Cimermancic P, Gulbahce N, Johnson JR, McGovern KE, et al (2012) Global landscape of HIV-human protein complexes. Nature 481: 365–370. 10.1038/nature10719 PMC3310911 22190034

[21] Sayers EW, Barrett T, Benson DA, Bolton E, Bryant SH, et al (2010) Database resources of the National Center for Biotechnology Information. Nucleic Acids Res 38: D5–16. 19910364 10.1093/nar/gkp967 PMC2808881

[22] Sargeant D, Deverasatty S, Luo Y, Baleta AV, Zobrist S, et al (2011) HIVToolbox, an integrated web application for investigating HIV. PloS One 6: e20122. 21647445 10.1371/journal.pone.0020122 PMC3102074

[23] Kagan R, Winters M, Merigan T, Heseltine P (2004) HIV type 1 genotypic resistance in a clinical database correlates with antiretroviral utilization. AIDS Res Hum Retroviruses 20: 1–9. 15000693 10.1089/088922204322749440

[24] Shafer R (2002) Genotypic Testing for Human Immunodeficiency Virus Type 1 Drug Resistance. Clinical Microbiology Reviews 15: 247–277. 11932232 10.1128/CMR.15.2.247-277.2002 PMC118066

[25] Vita R, Zarebski L, Greenbaum JA, Emami H, Hoof I, et al (2010) The immune epitope database 2.0. Nucleic Acids Res 38: D854–862. 19906713 10.1093/nar/gkp1004 PMC2808938

[26] Frankel AD, Chen L, Cotter RJ, Pabo CO (1988) Dimerization of the tat protein from human immunodeficiency virus: a cysteine-rich peptide mimics the normal metal-linked dimer interface. Proc Natl Acad Sci USA 85: 6297–6300. 2842763 10.1073/pnas.85.17.6297 PMC281956

[27] Auclair JR, Green KM, Shandilya S, Evans JE, Somasundaran M, et al (2007) Mass spectrometry analysis of HIV-1 Vif reveals an increase in ordered structure upon oligomerization in regions necessary for viral infectivity. Proteins 69: 270–284. 17598142 10.1002/prot.21471 PMC3366188

[28] DiMattia MA, Watts NR, Stahl SJ, Rader C, Wingfield PT, et al (2010) Implications of the HIV-1 Rev dimer structure at 3.2 A resolution for multimeric binding to the Rev response element. Proc Natl Acad Sci USA 107: 5810–5814. 20231488 10.1073/pnas.0914946107 PMC2851902

[29] Alfadhli A, Huseby D, Kapit E, Colman D, Barklis E (2007) Human immunodeficiency virus type 1 matrix protein assembles on membranes as a hexamer. J Virol 81: 1472–1478. 17108052 10.1128/JVI.02122-06 PMC1797500

[30] Singh P, Yadav GP, Gupta S, Tripathi AK, Ramachandran R, et al (2011) A novel dimer-tetramer transition captured by the crystal structure of the HIV-1 Nef. PLoS ONE 6: e26629. 22073177 10.1371/journal.pone.0026629 PMC3206816

[31] Venkatachari NJ, Walker LA, Tastan O, Le T, Dempsey TM, et al (2010) Human immunodeficiency virus type 1 Vpr: oligomerization is an essential feature for its incorporation into virus particles. Virol J 7: 119. 20529298 10.1186/1743-422X-7-119 PMC2894018

[32] Richter SN, Palù G (2006) Inhibitors of HIV-1 Tat-mediated transactivation. Curr Med Chem 13: 1305–1315. 16712471 10.2174/092986706776872989

[33] Pagans S, Sakane N, Schnölzer M, Ott M (2011) Characterization of HIV Tat modifications using novel methyl-lysine-specific antibodies. Methods 53: 91–96. 20615470 10.1016/j.ymeth.2010.07.001 PMC3478124

[34] Suhasini M, Reddy TR (2009) Cellular proteins and HIV-1 Rev function. Curr HIV Res 7: 91–100. 19149558 10.2174/157016209787048474

[35] Waldmann I, Spillner C, Kehlenbach RH (2012) The nucleoporin-like protein NLP1 (hCG1) promotes CRM1-dependent nuclear protein export. J Cell Sci 125: 144–154. 22250199 10.1242/jcs.090316

[36] He JJ, Henao-Mejia J, Liu Y (2009) Sam68 functions in nuclear export and translation of HIV-1 RNA. RNA Biol 6: 384–386. 19535902 10.4161/rna.6.4.8920 PMC2792590

[37] Descamps D, Lambert-Niclot S, Marcelin A-G, Peytavin G, Roquebert B, et al (2009) Mutations associated with virological response to darunavir/ritonavir in HIV-1-infected protease inhibitor-experienced patients. J Antimicrob Chemother 63: 585–592. 19147519 10.1093/jac/dkn544

[38] Colonno RJ, Thiry A, Limoli K, Parkin N (2003) Activities of atazanavir (BMS-232632) against a large panel of human immunodeficiency virus type 1 clinical isolates resistant to one or more approved protease inhibitors. Antimicrob Agents Chemother 47: 1324–1333. 12654666 10.1128/AAC.47.4.1324-1333.2003 PMC152527

[39] Colonno R, Rose R, McLaren C, Thiry A, Parkin N, et al (2004) Identification of I50L as the signature atazanavir (ATV)-resistance mutation in treatment-naive HIV-1-infected patients receiving ATV-containing regimens. J Infect Dis 189: 1802–1810. 15122516 10.1086/386291

[40] Yang B, Gao L, Li L, Lu Z, Fan X, et al (2003) Potent suppression of viral infectivity by the peptides that inhibit multimerization of human immunodeficiency virus type 1 (HIV-1) Vif proteins. J Biol Chem 278: 6596–6602. 12480936 10.1074/jbc.M210164200 PMC1350967

[41] Andréola ML (2009) Therapeutic potential of peptide motifs against HIV-1 reverse transcriptase and integrase. Curr Pharm Des 15: 2508–2519. 19601847 10.2174/138161209788682244

[42] Maes M, Loyter A, Friedler A (2012) Peptides that inhibit HIV-1 integrase by blocking its protein-protein interactions. FEBS J 279: 2795–2809. 22742518 10.1111/j.1742-4658.2012.08680.x

[43] Sluis-Cremer N, Tachedjian G (2002) Modulation of the oligomeric structures of HIV-1 retroviral enzymes by synthetic peptides and small molecules. Eur J Biochem 269: 5103–5111. 12392542 10.1046/j.1432-1033.2002.03216.x

[44] Bannwarth L, Rose T, Dufau L, Vanderesse R, Dumond J, et al (2009) Dimer disruption and monomer sequestration by alkyl tripeptides are successful strategies for inhibiting wild-type and multidrug-resistant mutated HIV-1 proteases. Biochemistry 48: 379–387. 19105629 10.1021/bi801422u

[45] Camarasa M-J, Velázquez S, San-Félix A, Pérez-Pérez M-J, Gago F (2006) Dimerization inhibitors of HIV-1 reverse transcriptase, protease and integrase: a single mode of inhibition for the three HIV enzymes? Antiviral Res 71: 260–267. 16872687 10.1016/j.antiviral.2006.05.021

[46] Root MJ, Kay MS, Kim PS (2001) Protein design of an HIV-1 entry inhibitor. Science 291: 884–888. 11229405 10.1126/science.1057453

[47] Malashkevich VN, Chan DC, Chutkowski CT, Kim PS (1998) Crystal structure of the simian immunodeficiency virus (SIV) gp41 core: conserved helical interactions underlie the broad inhibitory activity of gp41 peptides. Proc Natl Acad Sci USA 95: 9134–9139. 9689046 10.1073/pnas.95.16.9134 PMC21304

[48] Sarmady M, Dampier W, Tozeren A (2011) Sequence- and interactome-based prediction of viral protein hotspots targeting host proteins: a case study for HIV Nef. PLoS ONE 6: e20735. 21738584 10.1371/journal.pone.0020735 PMC3125164

[49] Baxter JD, Schapiro JM, Boucher CAB, Kohlbrenner VM, Hall DB, et al (2006) Genotypic changes in human immunodeficiency virus type 1 protease associated with reduced susceptibility and virologic response to the protease inhibitor tipranavir. J Virol 80: 10794–10801. 16928764 10.1128/JVI.00712-06 PMC1641746

[50] Sievers F, Wilm A, Dineen D, Gibson TJ, Karplus K, et al. (2011) Fast, scalable generation of high-quality protein multiple sequence alignments using Clustal Omega. Molecular Systems Biology 7. 10.1038/msb.2011.75 PMC3261699 21988835

[51] Briggs JAG, Kräusslich H-G (2011) The molecular architecture of HIV. J Mol Biol 410: 491–500. 21762795 10.1016/j.jmb.2011.04.021

[52] Robbins AH, Coman RM, Bracho-Sanchez E, Fernandez MA, Gilliland CT, et al (2010) Structure of the unbound form of HIV-1 subtype A protease: comparison with unbound forms of proteases from other HIV subtypes. Acta Crystallogr D Biol Crystallogr 66: 233–242. 20179334 10.1107/S0907444909054298 PMC2827345

[53] Mulky A, Kappes JC (2005) Analysis of human immunodeficiency virus type 1 reverse transcriptase subunit structure/function in the context of infectious virions and human target cells. Antimicrob Agents Chemother 49: 3762–3769. 16127051 10.1128/AAC.49.9.3762-3769.2005 PMC1195396

[54] Buxton P, Tachedjian G, Mak J (2005) Analysis of the contribution of reverse transcriptase and integrase proteins to retroviral RNA dimer conformation. J Virol 79: 6338–6348. 15858017 10.1128/JVI.79.10.6338-6348.2005 PMC1091692

[55] Meng X, Zhao G, Yufenyuy E, Ke D, Ning J, et al (2012) Protease cleavage leads to formation of mature trimer interface in HIV-1 capsid. PLoS Pathog 8: e1002886. 22927821 10.1371/journal.ppat.1002886 PMC3426514

[56] Chen K, Piszczek G, Carter C, Tjandra N (2013) The maturational refolding of the β-hairpin motif of equine infectious anemia virus capsid protein extends its helix α1 at capsid assembly locus. J Biol Chem 288: 1511–1520. 23184932 10.1074/jbc.M112.425140 PMC3548464

[57] Hagan NA, Fabris D (2007) Dissecting the protein-RNA and RNA-RNA interactions in the nucleocapsid-mediated dimerization and isomerization of HIV-1 stemloop 1. J Mol Biol 365: 396–410. 17070549 10.1016/j.jmb.2006.09.081 PMC1847390

[58] Frankel AD, Bredt DS, Pabo CO (1988) Tat protein from human immunodeficiency virus forms a metal-linked dimer. Science 240: 70–73. 2832944 10.1126/science.2832944

[59] Mao Y, Wang L, Gu C, Herschhorn A, Xiang S-H, et al (2012) Subunit organization of the membrane-bound HIV-1 envelope glycoprotein trimer. Nat Struct Mol Biol 19: 893–899. 22864288 10.1038/nsmb.2351 PMC3443289

[60] Horne WS, Johnson LM, Ketas TJ, Klasse PJ, Lu M, et al (2009) Structural and biological mimicry of protein surface recognition by alpha/beta-peptide foldamers. Proc Natl Acad Sci USA 106: 14751–14756. 19706443 10.1073/pnas.0902663106 PMC2736470

[61] Shu W, Liu J, Ji H, Radigen L, Jiang S, et al (2000) Helical interactions in the HIV-1 gp41 core reveal structural basis for the inhibitory activity of gp41 peptides. Biochemistry 39: 1634–1642. 10677212 10.1021/bi9921687

[62] Jenkins Y, Pornillos O, Rich RL, Myszka DG, Sundquist WI, et al (2001) Biochemical analyses of the interactions between human immunodeficiency virus type 1 Vpr and p6(Gag). J Virol 75: 10537–10542. 11581428 10.1128/JVI.75.21.10537-10542.2001 PMC114634

[63] Bourbigot S, Beltz H, Denis J, Morellet N, Roques BP, et al (2005) The C-terminal domain of the HIV-1 regulatory protein Vpr adopts an antiparallel dimeric structure in solution via its leucine-zipper-like domain. Biochem J 387: 333–341. 15571493 10.1042/BJ20041759 PMC1134961

[64] Sharpe S, Yau W-M, Tycko R (2006) Structure and dynamics of the HIV-1 Vpu transmembrane domain revealed by solid-state NMR with magic-angle spinning. Biochemistry 45: 918–933. 16411768 10.1021/bi051766k

[65] Solbak SMØ, Reksten TR, Hahn F, Wray V, Henklein P, et al (2013) HIV-1 p6 - a structured to flexible multifunctional membrane-interacting protein. Biochim Biophys Acta 1828: 816–823. 23174350 10.1016/j.bbamem.2012.11.010

[66] Techtmann SM, Ghirlando R, Kao S, Strebel K, Maynard EL (2012) Hydrodynamic and functional analysis of HIV-1 Vif oligomerization. Biochemistry 51: 2078–2086. 22369580 10.1021/bi201738a PMC3302947

